# Magnetic resonance cholangiopancreatography with compressed sensing at 1.5 T: clinical application for the evaluation of branch duct IPMN of the pancreas

**DOI:** 10.1007/s00330-020-06996-2

**Published:** 2020-06-18

**Authors:** Benjamin Henninger, Michael Steurer, Michaela Plaikner, Elisabeth Weiland, Werner Jaschke, Christian Kremser

**Affiliations:** 1 Department of Radiology, Medical University of Innsbruck, Anichstraße 35, 6020 Innsbruck, Austria; 2 Siemens Healthcare GmbH, Henkestraße 127, 91052 Erlangen, Germany

**Keywords:** Magnetic resonance imaging, Pancreas, Pancreatic intraductal neoplasms

## Abstract

**Objectives:**

To evaluate magnetic resonance cholangiopancreatography (MRCP) with compressed sensing (CS) for the assessment of branch duct intraductal papillary mucinous neoplasm (BD-IPMN) of the pancreas. For this purpose, conventional navigator-triggered (NT) sampling perfection with application-optimized contrast using different flip angle evolutions (SPACE) MRCP was compared with various CS-SPACE-MRCP sequences in a clinical setting.

**Methods:**

A total of 41 patients (14 male, 27 female, mean age 68 years) underwent 1.5-T MRCP for the evaluation of BD-IPMN. The MRCP protocol consisted of the following sequences: conventional NT-SPACE-MRCP, CS-SPACE-MRCP with long (BHL, 17 s) and short single breath-hold (BHS, 8 s), and NT-CS-SPACE-MRCP. Two board-certified radiologists evaluated image quality, duct sharpness, duct visualization, lesion conspicuity, confidence, and communication with the main pancreatic duct in consensus using a 5-point scale (1–5), with higher scores indicating better quality/delineation/confidence. Maximum intensity projection reconstructions and originally acquired data were used for evaluation. Wilcoxon signed-rank test was used to compare the intra-individual difference between sequences.

**Results:**

BHS-CS-SPACE-MRCP had the highest scores for image quality (3.85 ± 0.79), duct sharpness (3.81 ± 1.05), and duct visualization (3.81 ± 1.01). There was a significant difference compared with NT-CS-SPACE-MRCP (*p* < 0.05) but no significant difference to the standard NT-SPACE-MRCP (*p* > 0.05). Concerning diagnostic quality, BHS-CS-SPACE-MRCP had the highest scores in lesion conspicuity (3.95 ± 0.92), confidence (4.12 ± 1.08), and communication (3.8 ± 1.06), significantly higher compared with NT-SPACE-MRCP, BHL-SPACE-MRCP, and NT-CS-SPACE-MRCP (*p* = <0.05).

**Conclusions:**

MRCP with CS 3D SPACE for the evaluation of BD-IPMN at 1.5 T provides the best results using a short breath-hold sequence. This approach is feasible and an excellent alternative to standard NT 3D MRCP sequences.

**Key Points:**

*• 1.5-T MRCP with compressed sensing for the evaluation of branch duct IPMN is a feasible method.*

*• Short breath-hold sequences provide the best results for this purpose.*

## Introduction

Magnetic resonance imaging (MRI) is the method of choice for the evaluation of cystic pancreatic lesions [1]. Among all cystic lesions, the intraductal papillary mucinous neoplasm (IPMN) of the pancreas is increasingly the focus of attention as many of these cystic lesions are discovered incidentally [2]. MRI is mainly used for its non-invasive diagnosis, especially for the branch duct–type IPMN for detecting communication with the main pancreatic duct. Magnetic resonance cholangiopancreatography (MRCP), based on heavily T2-weighted sequences, is therefore the cornerstone and an established method [3–5]. Normally, thin-slice, isotropic, navigator-triggered (NT) 3D MRCP sequences are performed to prove ductal communication. Single-shot breath-hold 2D sequences are also frequently used, but their benefit of short acquisition time is often limited due to thicker sections and therefore the missing availability of crucial 3D reconstructions [6].

The management of IPMN is constantly evolving with currently two main strategies: the Fukuoka guidelines and the white paper of the ACR Incidental Findings Committee [2, 7]. With possible follow-up intervals of 6 months over a period of 2 years, it is important that the MRI examination is optimized for these patients. Standard and widely established MRCP protocols of the pancreas normally have long acquisition times; especially, the NT 3D MRCP sequences can take up to 7 min, strongly depending on the cooperation (regular respiratory rhythm) of the patient [8]. Furthermore, such sequences may also result in suboptimal image quality in patients who do not have a consistent breathing pattern.

Compressed sensing (CS) is a relatively recent approach for the acceleration of image acquisition and is used in different anatomic regions [9–11]. It uses a sparse, incoherent undersampling of k-space and a nonlinear iterative reconstruction to correct for subsampling artifacts. This can preserve image quality and enables the acquisition of fast 3D MRCP datasets, even in a single breath-hold [12, 13]. Although the benefits of CS are obvious, it is important to evaluate such accelerated sequences for daily clinical routine with emphasize on robustness and diagnostic accuracy. In current literature, the use of CS for MRCP has already frequently been reported, mainly at 3 T [12–14]. Nearly all studies found the CS approach feasible with comparable results to standard MRCP sequences, which have significantly longer examination time.

Our purpose was to evaluate MRCP with CS for the assessment of IPMN of the pancreas at 1.5 T. For this purpose, conventional navigator-triggered (NT) sampling perfection with application-optimized contrast using different flip angle evolutions (SPACE) 3D MRCP was compared with various prototype 3D CS-SPACE-MRCP sequences in a clinical setting.

## Materials and methods

Written informed consent on the MRI was obtained from each patient. Institutional review board approval was granted by means of a general waiver for studies with retrospective data analysis (local research ethics committee, Medical University of Innsbruck; 20 February 2009). The authors who are employees of Siemens Healthcare had no control of any data and were not involved in the execution of the study.

### Patients

**Fig. 1:**
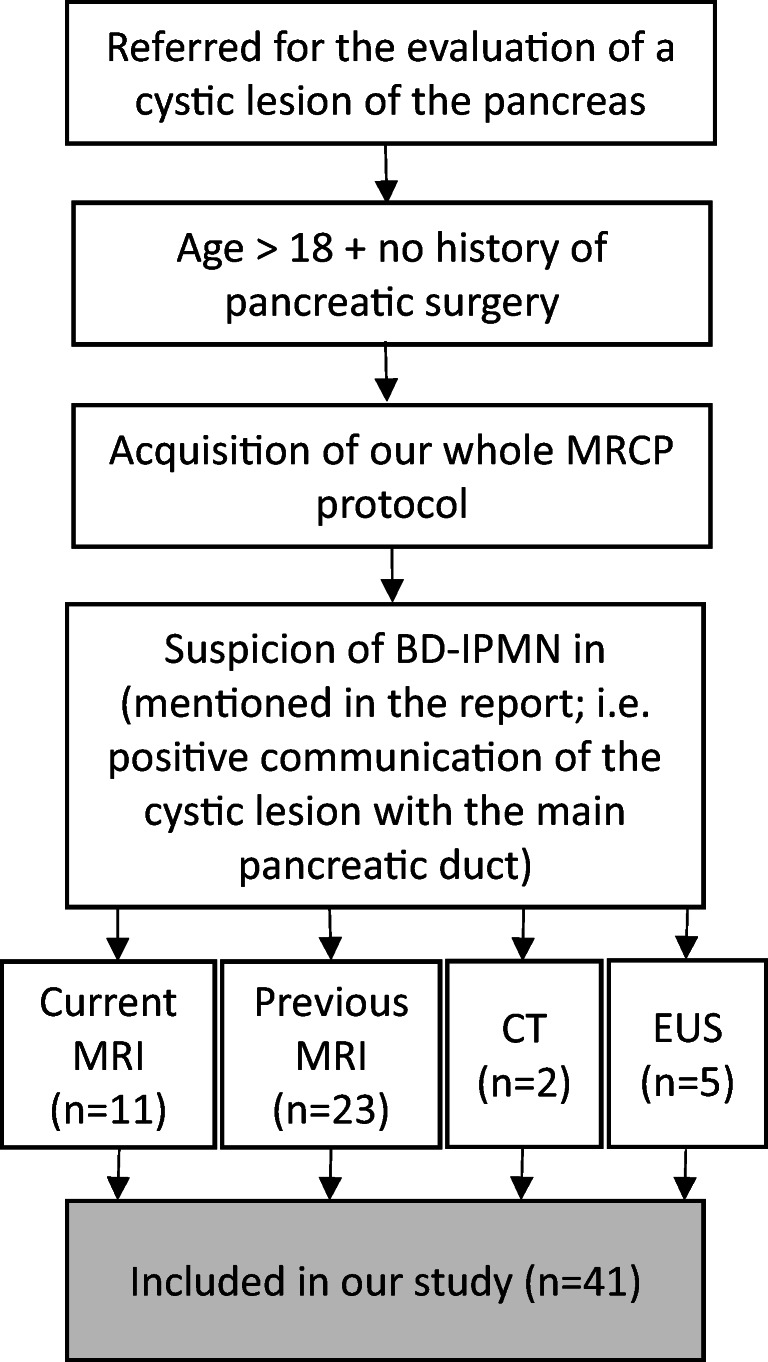
Overview of patient inclusion criteria, visualized as a flow chart

Patients were included into our study using the following inclusion criteria: (1) patients referred to the Department of Radiology (Medical University of Innsbruck) for the MRI evaluation of a cystic lesion of the pancreas, (2) age > 18 years, (3) no history of pancreatic surgery, (4) acquisition of our whole MRCP protocol as mentioned below, (5) suspicion of branch duct IPMN (BD-IPMN) in our current MRI report (positive communication of the cystic lesion with the main pancreatic duct in at least one sequence, no previous MRI available) or in other imaging procedures (e.g., computed tomography (CT), endoscopic ultrasound (EUS), or previous MRI), i.e., positive communication of the cystic lesion with the main pancreatic duct, mentioned in the report (see Fig. 1).

After searching our internal database, we retrospectively included 41 patients (27 female; 14 male; median age, 69 years; range, 46–89 years) between March 2018 and December 2018. Thereby, 18/41 were referred with suspicion and 23/41 for follow-up of BD-IPMN (=patients with previous MRI).

### MR imaging

All studies were performed on a 1.5-T MR system (MAGNETOM Avanto^fit^, Siemens Healthcare) using an 18-element body matrix coil and a 32-element spine coil. Patients were scanned in supine position. Standard examination preparation for each patient included oral administration of 200 mL of water with one tablet of Supradyn forte (Bayer) 10 min prior to the examination, serving as a negative contrast agent. The MRCP protocol consisted of the following sequences in coronal orientation: (1) conventional navigator-triggered (NT) sampling perfection with application-optimized contrast using different flip angle evolutions (SPACE) 3D MRCP (NT-SPACE-MRCP); (2) prototype compressed sensing SPACE-MRCP with a long single breath-hold of 17 s (BHL-CS-SPACE-MRCP); (3) prototype compressed sensing SPACE-MRCP with a short single breath-hold of 8 s (BHS-CS-SPACE-MRCP); (4) prototype navigator-triggered, compressed sensing SPACE-MRCP (NT-CS-SPACE-MRCP). Details of these four sequences are provided in Table 1. Maximum intensity projection (MIP) was performed for all four sequences on the satellite console of the MR unit.

**Table 1:** Sequence parameters (all sequences were acquired in coronal orientation)

	NT-SPACE-MRCP	BHL-CS-SPACE-MRCP	BHS-CS-SPACE-MRCP	NT-CS-SPACE-MRCP
TR (ms)	Variable depending on the respiratory rate	2000	1700	Variable depending on the respiratory rate
TE (ms)	698	551	433	696
Echo train length	270	162	230	180
Flip angle (°)	140	140	140	140
Acquisition matrix	353 × 384	134 × 320	115 × 320	128 × 320
Phase encoding direction	R ≫ L	R ≫ L	R ≫ L	R ≫ L
Acceleration factor	GRAPPA 2	CS 26	CS 26	CS 19
CS k-space data sampling (%)	-	3.8	3.8	5.4
Slice thickness (mm)	1	1.2	1.2	1
FOV (mm^2^)	320 × 320	210 × 400	210 × 400	200 × 400
Acquisition time (min)	Variable: mean 05:20; max. 08:17; min. 02:53	00:17 (breath-hold)	00:08 (breath-hold)	Variable: mean 02:13; max. 04:06; min. 01:32

In addition, our standard sequences for imaging the pancreas, including T1- and T2-weighted sequences, diffusion-weighted imaging, and contrast-enhanced dynamic scans, were performed for all patients. These sequences were not utilized for the present data analysis.

For CS-MRCP, a prototype 3D SPACE sequence was used with incoherent undersampling and CS reconstruction. Incoherent undersampling was obtained using a Poisson-Disk pattern [12]. For BHL-CS-SPACE-MRCP and BHS-CS-SPACE-MRCP, 3.8% k-space data sampling was chosen leading to an acceleration factor of 26. For NT-CS-SPACE-MRCP, the acceleration factor was 19 (5.4% k-space data sampling). CS reconstruction was automatically performed in-line after image acquisition using a fast iterative soft-thresholding algorithm (20 iterations) together with regularization. Parameters for CS reconstruction were identical for all CS sequences.

### Image analysis

All MRI scans were assessed independently by 2 radiologists with 12 years (B.H.) and 8 years (M.P.) of experience in reading MRI images of the abdomen.

Image analysis was performed in four separate reading sessions with 4-week interval to reduce potential recall bias. The four available MRCP sequences of each anonymized patient were randomized; one sequence per patient was then evaluated in one session. No data from the other performed sequences were made available during the image analysis. Readers graded all four sequences separately on a 5-point Likert-type scale in the following categories: image quality, duct sharpness, duct visualization, lesion conspicuity, confidence, and communication. In general, for the diagnostic approach (lesion conspicuity, confidence, and communication), the presence of a cystic lesion in communication with the main pancreatic duct was assessed, with higher scores indicating better results (Table 2). In case of multiple cystic lesion (> 1), only the largest lesion was evaluated. MIPs and the source images were evaluated together.

**Table 2:** Parameter scores of image analysis

Score	Image quality	Duct sharpness	Duct visualization	Lesion conspicuity	Confidence	Communication
1	Non-diagnostic	Non-diagnostic	No visualization	Unreadable	Definitely absent	Unreadable images
2	Poor	Substantial blur	Poorly visualized, limited diagnostic value	Poor	Probably absent	Indeterminate
3	Fair/acceptable	Mild blur with mild image quality degradation	Partial or blurry, decreased image quality	Acceptable	Indeterminate	Poor depiction
4	Good	No or minimal blur	Well visualized	Good	Probably present	Good depiction
5	Excellent	No blur	Excellently visualized	Excellent	Definitely present	Excellent depiction

Finally, a consensus between both radiologists was built in a second session.

### Statistical analysis

Descriptive statistics were used including mean or median, standard deviation, and range to present patient data, acquisition time, and scoring results. All statistical calculations were performed using R Project for Statistical Computing [15]. Comparison of acquisition time and scoring between different sequences was performed using a Wilcoxon signed-rank test. To assess inter-rater agreement, the Cohen’s kappa coefficient (0.21–0.40, fair agreement; 0.41–0.60, moderate agreement; 0.61–0.80, good agreement; 0.81–1.00, perfect agreement) with equal weights was calculated using the irr-package for R [16]. Results were considered statistically significant for *p* < 0.05.

## Results

All results are summarized in Table 3.

**Table 3:** Results (mean score ± standard deviation)

Sequence	Image quality	Duct sharpness	Duct visualization	Lesion conspicuity	Confidence	Communication
NT-SPACE	3.56 ± 0.95	3.46 ± 1.05	3.49 ± 1.05	3.56 ± 0.90	3.71 ± 0.93	3.44 ± 0.90
BHL-CS-SPACE	3.56 ± 0.87	3.56 ± 1.03	3.49 ± 1.03	3.59 ± 1.07	3.66 ± 1.11	3.51 ± 1.12
BHS-CS-SPACE	3.85 ± 0.79	3.81 ± 1.05	3.81 ± 1.01	3.95 ± 0.92	4.12 ± 1.08	3.98 ± 1.06
NT-CS-SPACE	3.22 ± 0.85	3.15 ± 1.09	3.10 ± 0.94	3.29 ± 0.98	3.32 ± 1.13	3.17 ± 1.09

### Acquisition time

Mean acquisition time for NT-SPACE was 05:20 min (range, 02:53–08:17 min) and 02:13 min (range, 01:32–04:06 min) for the NT-CS-SPACE. The difference in acquisition time between both sequences was highly significant (*p* < 0.001). The CS BH sequences had fixed acquisition times (one breath-hold) of 17 s and 8 s (BHL-CS-SPACE and BHS-CS-SPACE).

### Image quality

Image quality scores for BHS-CS-SPACE (3.85 ± 0.79) were higher than for all other evaluated sequences, with a significant difference only to NT-CS-SPACE (3.22 ± 0.85; *p* < 0.05) and BHL-CS-SPACE (3.56 ± 0.87; *p* < 0.05). NT-SPACE (3.56 ± 0.95) revealed a significantly higher score (*p* < 0.05) than NT-CS-SPACE (3.22 ± 0.85).

For duct sharpness and duct visualization, BHS-CS-SPACE reached the highest score (3.81 ± 1.05 and 3.81 ± 1.01, respectively) with a significant difference (for both) only to NT-CS-SPACE (*p* < 0.05).

For image quality, duct sharpness, and duct visualization, the difference between the NT-SPACE (our current standard sequence) and the BHS-CS-SPACE (with the highest scores) was not significant (*p* > 0.05). The NT-CS-SPACE sequence had the lowest scores among all evaluated sequences.

### Diagnostic quality

The BHS-CS-SPACE sequence had the highest scores in lesion conspicuity (3.95 ± 0.92), confidence (4.12 ± 1.08), and communication (3.98 ± 1.06). All scores were significantly higher compared with NT-SPACE, BHL-CS-SPACE, and NT-CS-SPACE (*p* < 0.05).

Overall, NT-CS-SPACE reached the lowest scores concerning diagnostic quality; compared with the standard NT-SPACE, statistical significance was not reached in lesion conspicuity, confidence, and communication (*p* > 0.05).

**Fig. 2:**
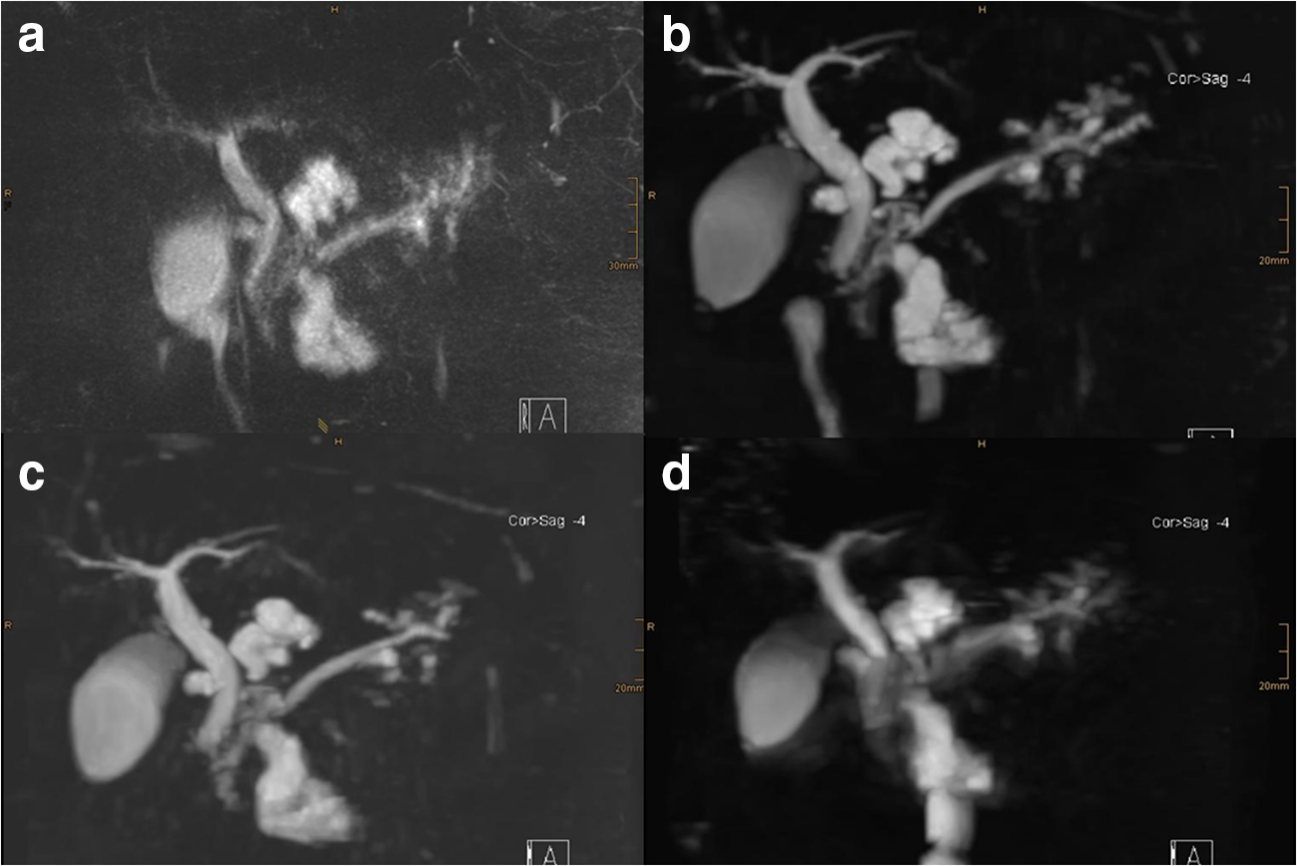
A 71-year-old female patient with multiple BD-IPMN. **a** Conventional NT-SPACE-MRCP providing the worst quality with the longest acquisition time (04:13 min). CS-SPACE-MRCP with breath-hold shows the best image quality (image **b** is BHL and image **c** is BHS). **d** NT-CS-SPACE-MRCP with 01:32-min acquisition time also provides only average image quality. Scores were as follows [image quality/duct sharpness/duct visualization/lesion conspicuity/confidence/communication]: (**a**) NT-SPACE [2/2/2/2/3/2]; (**b**) BHL-CS-SPACE [4/4/4/4/5/4]; (**c**) BHS-CS-SPACE [4/5/4/4/5/5]; (**d**) NT-CS-SPACE [3/3/2/2/2/3] (MIPs are displayed; windowing was adjusted for optimal visualization)

**Fig. 3:**
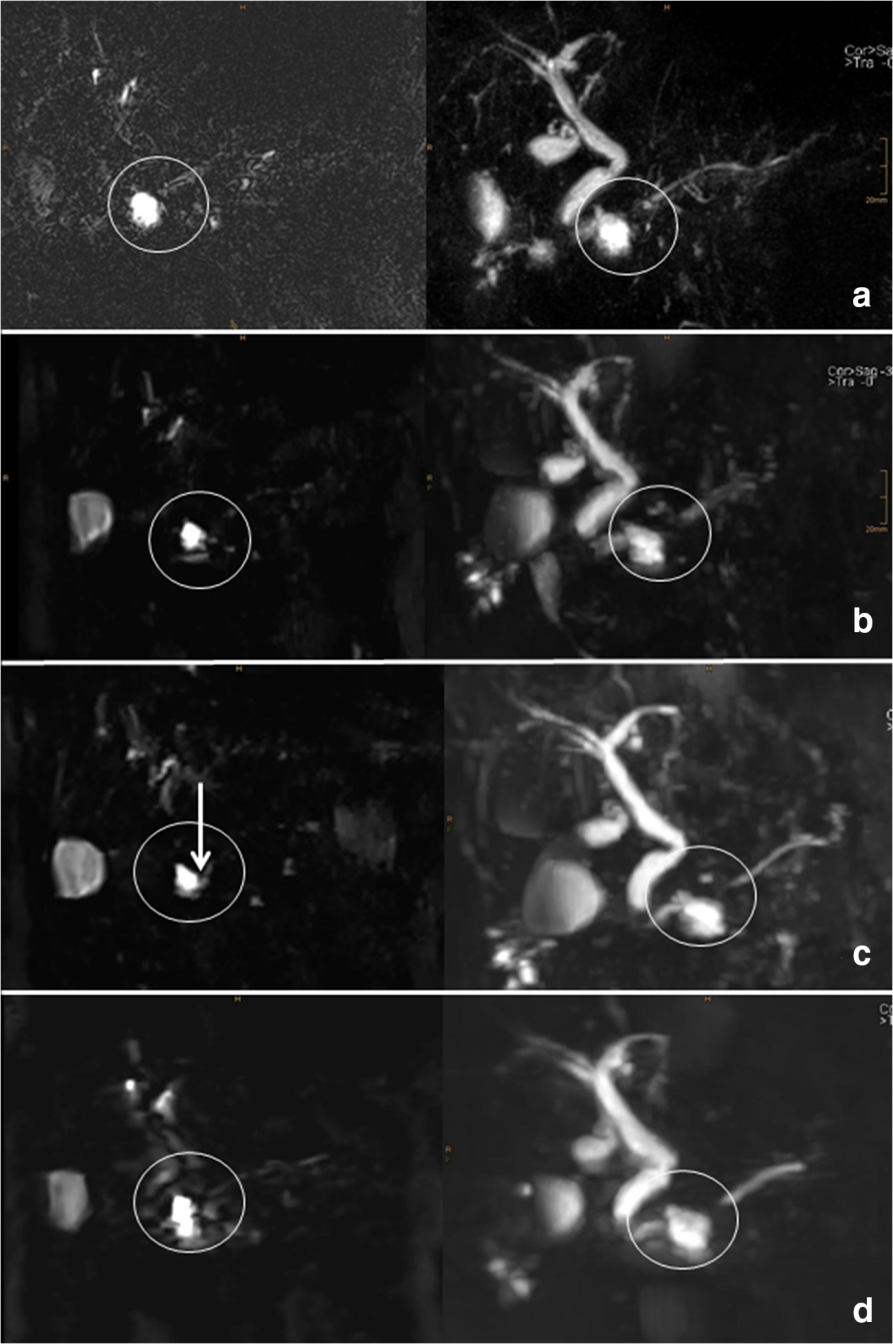
A 56-year-old male patient with BD-IPMN. Difference between the sequences for source images (left) and MIPs (right) are shown. **a** Conventional NT-SPACE-MRCP; the communication between the cystic lesion and the pancreatic duct cannot be visualized (circles). It can only barely be depicted with BHL-CS-SPACE-MRCP (circles, **b**). **c** BHS-CS-SPACE-MRCP was able to illustrate the communication in the source images (arrow) which made the diagnosis of a BD-IPMN possible. **d** NT-CS-SPACE-MRCP showed acceptable image quality, but the communication was also hard to depict due to blurring. Scores were as follows [image quality/duct sharpness/duct visualization/lesion conspicuity/confidence/communication]: (**a**) NT-SPACE [4/3/3/2/2/1]; (**b**) BHL-CS-SPACE [3/1/2/2/3/2]; (**c**) BHS-CS-SPACE [4/4/4/4/4/4]; (**d**) NT-CS-SPACE [3/3/2/3/3/3]

Examples are provided in Figs. 2 and 3.

### Inter-rater agreement

Inter-rater agreement was perfect for image quality, duct sharpness, duct visualization, and lesion conspicuity (weighted *k* = 0.826–0.915, *p* = < 0.05) and good to perfect for confidence and communication (weighted *k* = 0.715–0.862, *p* = < 0.05) (see Table 4 for detailed information).

**Table 4:** Inter-rater agreement

Sequence	Image quality	Duct sharpness	Duct visualization	Lesion conspicuity	Confidence	Communication
NT-SPACE	0.874 (0.86−0.89	0.914 (0.90−0.93)	0.855 (0.84−0.87)	0.897 (0.89−0.91)	0.78 (0.76−0.80)	0.759 (0.74−0.78)
BHL-CS-SPACE	0.896 (0.88−0.91)	0.869 (0.85−0.89)	0.856 (0.84−0.87)	0.892 (0.88−0.91)	0.756 (0.73−0.78)	0.84 (0.82−0.86)
BHS-CS-SPACE	0.885 (0.87−0.89)	0.915 (0.90−0.93)	0.886 (0.87−0.90)	0.851 (0.84−0.86)	0.746 (0.72−0.77)	0.862 (0.85−0.88)
NT-CS-SPACE	0.86 (0.85−0.87)	0.877 (0.86−0.89)	0.826 (0.81−0.84)	0.832 (0.82−0.85)	0.715 (0.69−0.74)	0.837 (0.82−0.86)

*p* value was < 0.05 for all evaluations; numbers in parentheses are 95% confidence interval

## Discussion

In our study, the BHS-SPACE-CS-MRCP sequence showed the highest scores concerning image and diagnostic quality for the evaluation of BD-IPMN. Interestingly, results from BHS-SPACE-CS were noticeably better compared with the NT-SPACE-CS with significantly better results concerning all evaluated categories. The main advantage is the short acquisition time with just one very short breath-hold which also seems to influence the image quality and diagnostic confidence positively for CS-MRCP sequences at 1.5 T.

The diagnosis of the BD-IPMN is based on the proof of a communication with the main pancreatic duct. In general, this preferably is performed with thin-slice 3D sequences with longer acquisition time compared with 2D sequences [17]. We are aware that the communication with the main pancreatic duct is not always detectable with MRI in general. However, it was not the aim of our study to evaluate the diagnostic accuracy of MRCP for the diagnosis of BD-IPMN but to find the best possible and optimized MRI sequence for this purpose. In this respect, a BH-SPACE-CS sequence with a very short breath-hold seems to be an ideal sequence for the evaluation of BD-IPMN. Especially if follow-up is needed, this further reduces the required scanning time without loss of diagnostic value.

Chandarana et al compared a breath-hold accelerated SPACE sequence with sparsity-based iterative reconstruction against a conventional respiratory-triggered (RT)-SPACE-MRCP at a 3-T system [12]. The overall image quality scores for BH SPARSE-SPACE were higher than those for RT-SPACE, and it was concluded that a BH sequence showed similar or superior image quality for the pancreatic duct, despite 17-fold shorter acquisition time. This study group was one of the first to evaluate CS techniques for MRCP, and their results are comparable with ours. Most of the other so far published studies in recent literature found comparable (or even superior) results between 3D MRCP sequences performed with and without CS at 3 T; in all cases, a shortening of the examination time with constant to better image quality was reported [13, 14, 18].

Furlan et al also assessed 3D CS-MRCP for the evaluation of pancreatic cystic lesions [19]. Their study, however, had no emphasis on IPMN and was carried out on a 3-T scanner, and the evaluated CS sequence was respiratory- or navigator-triggered. The acquisition time was decreased twofold by using CS without significantly compromising the overall image quality and the degree of artifacts.

There is little literature on the evaluation of CS-MRCP sequences at 1.5 T. The study by Taron et al evaluated CS-MRCP sequences with 1.5 T and 3 T [20]. In addition to a phantom study, they evaluated 66 patients (19 for IPMN) with a conventional 3D NT-SPACE and a prototype 3D NT/BH CS-SPACE. The BH CS-SPACE resulted in significantly inferior ratings in all aspects compared with NT-CS-SPACE, and the NT-CS-SPACE was found superior to the standard NT-SPACE. These results are in contradiction to ours. We had no comparison to 3-T images, but, in our patient cohort, the CS BH sequences were superior to CS NT sequences. Further CS NT sequences had the most inferior ratings of all tested sequences. One reason for the different results could possibly be seen in the different approach of rating the sequences. In our study, the main focus was on the representation of the pancreatic duct, and we did not evaluate, e.g., the visibility of peripheral structures in the biliary system. Another reason for our good results with the CS BH sequence might also be our relatively old patient population (mean age 68 years; range, 46–89 years). Especially for these patients, the very low breath-hold time of only 7 s might be beneficial. It has been shown that short examination time in general leads to better image quality [21]. It has to be noted that in agreement with our results, other studies as well found better results with the BH CS sequences at 3 T, compared with NT CS sequences [22, 23].

Tokoro et al found that the BH CS-MRCP sequence brings added value to standard NT MRCP, because it compensates the image deterioration of NT MRCP [24]. In detail, they found 43/113 cases with clinically inadequate image quality with the NT MRCP. The image quality in 13/43 cases could be improved to clinically adequate by adding the BH CS-MRCP sequence.

Image reconstruction for the CS sequences is usually very long (approx. 5 min) [17]. By integrating the CS sequences as the last sequence into our standard protocol, the time of patient change was used for reconstruction without delaying the routine workflow. With this approach, however, there is no possibility to check image quality and to eventually rerun the sequence, as the images were only available when the next patient was already in the examination room. The time of image reconstruction was not further evaluated in our study, because our MR scanner was equipped with the standard hardware which was not optimized to reconstruct demanding compressed sensing applications.

There are limitations to this study. First, this is a retrospective study. Second, we did not perform correlation with concrete pathology; e.g., the diagnosis of BD-IPMN was not further assessed nor was there any correlation with other diagnostic procedures. Our study aimed at finding an ideal sequence and comparing the performance between several sequences. Third, we had no records of patient cooperation. This might be of interest as we hypothesized that the age could be a relevant factor for image quality which was not proven or correlated with the general condition of our patients. Finally, our study population was rather small.

## Conclusion

MRCP imaging with CS-SPACE for the evaluation of BD-IPMN at 1.5 T provides the best results using a short breath-hold sequence with results comparable with conventional NT-SPACE and superior to NT-CS-SPACE. This approach is feasible and an excellent alternative to standard 3D NT-MRCP sequences, especially concerning the diagnosis and follow-up of BD-IPMN.

## Abbreviations

CS: Compressed sensing
IPMN: Intraductal papillary mucinous neoplasm
MIP: Maximum intensity projection
MRCP: Magnetic resonance cholangiopancreatography
MRI: Magnetic resonance imaging
NT: Navigator-triggered
SPACE: Sampling perfection with application-optimized contrast using different flip angle evolutions
